# Supplementary material for: Generation of machine-learning derived cancer vulnerability indicator to determine the spatial burden of cancer outcomes
Source: PLoS One. 2026 Feb 20;21(2):e0319539. doi: 10.1371/journal.pone.0319539 (PMC12923003; doi:10.1371/journal.pone.0319539)
Supplement: S1 Appendice — (DOCX) [file pone.0319539.s001.docx]

# Appendix 1 Modelling of indirect standardized incidence ratios

The outcome of interest was the smoothed indirect standardized incidence ratios (SIR) of lung cancer for the periods of 2016-2019 in Queensland. The models for estimating SIRs has been reported elsewhere [1]. Briefly, SIRs are the ratio of the modelled number of lung cancer cases to the expected number of cases, assuming the age-specific incidence rate were the same as the Queensland average. The expected number of lung cancer cases in area $i$ ($E_{i}$) was calculated as:

$$E_{i}=\sum_{k=1}^{K} \frac{N_{k}}{M_{k}}m_{ik}$$

where:

$N_{k}$ is the number of lung cancer cases in Queensland for age group $k$;

$M_{k}$ is the total Queensland population for age group $k$;

$m_{ik}$ is the population in area $i$ for age group $k$;

Age group represents 5-year age groups from age 20-24 to 80-84 and 85+.

Bayesian spatial incidence models were used to estimate smoothed SIRs for each SA2, maintaining confidentiality and distinguishing spatial patterns from random variation [2-5]. The models account for spatial dependencies in the underlying disease rates that arise from the first law of geography, which states that areas near each other are likely to be more similar than areas that are distant from each other {Tobler, 1970 #72}.

A Besag-Yorke-Mollié (BYM) prior was applied to the spatial random effect [6]. Using the BYM conditional autoregressive (CAR) prior, the spatial effect for each area has two separate terms: one describing the spatially structured component of the area-level variation and another describing the unstructured area-level variation [6]. The models were fitted using Markov Chain Monte Carlo (MCMC) simulation [8], generating a posterior probability distribution for each parameters.

The Bayesian spatial BYM model for incidence can be written as:

$$y_{i} \sim\mathrm{Poisson}(E_{i}\lambda_{i})$$

$$\log\left( \lambda_{i} \right)= \beta+S_{i}+e_{i}$$

$$\beta\sim N(0,100000)$$

$$E_{i}=\sum_{k=1}^{K} \frac{n_{k}}{pop_{k}}pop_{ik}$$

$$S_{i}|S_{\backslash i} \sim N\left( \frac{\Sigma_{h}w_{ih}S_{h}}{\Sigma_{h}w_{ih}}, \frac{\sigma_{S}^{2}}{\Sigma_{h}w_{ih}} \right)$$

$$\sigma_{S}^{2} \sim\mathrm{InverseGamma}(1, 0.01)$$

$$e_{i} \sim N(0, \sigma_{e}^{2})$$

$$\sigma_{e}^{2} \sim\mathrm{InverseGamma}(1, 0.01)$$

where:

$y_{i}$ is the number of diagnoses observed in area $i$;

$E_{i}$ is the expected number of counts, as described in Appendix 1;

$S_{i}$ is the spatial random effect parameter of the intrinsic CAR (ICAR) model;

$e_{i}$ is the unstructured random effect for area $i$;

$n_{k}$ is the number of cases in Queensland for age group $k;$

$pop_{k}$ is the total Queensland population for age group $k;$

$pop_{ik}$ is the population in area $i$ for age group $k$;

$w_{ih}$ is the (i,h)th element of the spatial weights matrix $W$, in which each area is represented by a unique row and column. The spatial weights matrix was a binary, first-order adjacency matrix. i.e., $w_{ih}$ = 1 if area $i$ shares a boundary with area $h$ and 0 otherwise. Moreover, $w_{ih}$ = 0 if $i$ = $h$.

Modelled estimates were indirectly age-adjusted since $E_{i}$ considers the age structure and population size.

Following the Australian Cancer Atlas 2.0 [10], the point estimate of the standardised incidence ratio (SIR) was taken to be the posterior median of $exp(\lambda_{i})$. The exceedance probability ($EP_{i}$) posterior probability ($PP_{i}$) were calculated as the number of MCMC iterations where the SIR was greater than 1:

$$EP_{i}=\frac{\sum_{q=1}^{Q} I_{\left\{ SIR_{iq}>1 \right\}}}{Q}$$

where $Q$ is the total number of MCMC iterations and $I_{\left\{ SIR_{iq}>1 \right\}}$ for area $i$ and MCMC iteration $q$ was 1 if $SIR_{iq}$ was greater than 1 and 0 otherwise.

# Appendix 2 Smoothed lung cancer standardized incidence ratios and exceedance probability for 519 SA2 areas in Queensland

| SA2 Code | SA2 Name | Standardised Incidence Ratio (SIR) | Exceedance Probability (EP) |
| --- | --- | --- | --- |
| 31001 | Alexandra Hills | 1.00 | 0.49 |
| 31002 | Belmont - Gumdale | 0.87 | 0.18 |
| 31003 | Birkdale | 0.95 | 0.34 |
| 31004 | Capalaba | 0.96 | 0.37 |
| 31005 | Thorneside | 1.09 | 0.68 |
| 31006 | Wellington Point | 0.70 | 0.01 |
| 31007 | Cleveland | 0.97 | 0.39 |
| 31008 | Ormiston | 0.82 | 0.11 |
| 31009 | Redland Bay | 0.86 | 0.13 |
| 31011 | Sheldon - Mount Cotton | 0.88 | 0.20 |
| 31012 | Thornlands | 0.80 | 0.05 |
| 31013 | Victoria Point | 0.88 | 0.13 |
| 31014 | Brisbane Port - Lytton | 0.93 | 0.38 |
| 31015 | Manly - Lota | 1.10 | 0.73 |
| 31016 | Manly West | 0.88 | 0.18 |
| 31017 | Murarrie | 0.92 | 0.31 |
| 31018 | Tingalpa | 1.10 | 0.76 |
| 31019 | Wakerley | 0.94 | 0.35 |
| 31020 | Wynnum | 0.84 | 0.11 |
| 31021 | Wynnum West - Hemmant | 1.05 | 0.66 |
| 31022 | Bald Hills | 0.89 | 0.21 |
| 31023 | Bridgeman Downs | 0.69 | 0.01 |
| 31024 | Carseldine | 0.82 | 0.08 |
| 31025 | Everton Park | 0.75 | 0.03 |
| 31026 | McDowall | 0.72 | 0.01 |
| 31027 | Aspley | 0.77 | 0.02 |
| 31028 | Chermside | 1.00 | 0.51 |
| 31029 | Chermside West | 0.72 | 0.02 |
| 31030 | Geebung | 0.79 | 0.07 |
| 31031 | Kedron - Gordon Park | 0.80 | 0.06 |
| 31032 | Stafford | 0.90 | 0.25 |
| 31033 | Stafford Heights | 0.74 | 0.03 |
| 31034 | Wavell Heights | 0.79 | 0.06 |
| 31035 | Boondall | 0.73 | 0.02 |
| 31036 | Brisbane Airport | 0.82 | 0.15 |
| 31037 | Eagle Farm - Pinkenba | 0.74 | 0.08 |
| 31038 | Northgate - Virginia | 0.78 | 0.07 |
| 31039 | Nudgee - Banyo | 0.68 | 0.01 |
| 31040 | Nundah | 0.79 | 0.05 |
| 31041 | Bracken Ridge | 0.96 | 0.37 |
| 31042 | Brighton (Qld) | 0.92 | 0.27 |
| 31043 | Deagon | 0.92 | 0.32 |
| 31044 | Sandgate - Shorncliffe | 0.84 | 0.13 |
| 31045 | Taigum - Fitzgibbon | 0.98 | 0.43 |
| 31046 | Zillmere | 0.97 | 0.42 |
| 31047 | Camp Hill | 0.86 | 0.16 |
| 31048 | Cannon Hill | 0.98 | 0.46 |
| 31049 | Carina | 0.71 | 0.01 |
| 31050 | Carina Heights | 0.91 | 0.28 |
| 31051 | Carindale | 0.77 | 0.02 |
| 31052 | Annerley | 0.83 | 0.11 |
| 31053 | Coorparoo | 0.92 | 0.27 |
| 31054 | Fairfield - Dutton Park | 1.06 | 0.62 |
| 31055 | Greenslopes | 1.00 | 0.49 |
| 31056 | Holland Park | 0.97 | 0.41 |
| 31057 | Holland Park West | 0.97 | 0.43 |
| 31058 | Woolloongabba | 0.92 | 0.30 |
| 31059 | Yeronga | 0.77 | 0.03 |
| 31060 | Eight Mile Plains | 0.92 | 0.26 |
| 31061 | Macgregor (Qld) | 0.93 | 0.33 |
| 31062 | Mansfield (Qld) | 0.76 | 0.03 |
| 31063 | Mount Gravatt | 1.02 | 0.57 |
| 31064 | Rochedale - Burbank | 0.84 | 0.12 |
| 31065 | Upper Mount Gravatt | 0.94 | 0.32 |
| 31066 | Wishart | 0.80 | 0.06 |
| 31067 | Coopers Plains | 1.01 | 0.53 |
| 31068 | Moorooka | 0.98 | 0.44 |
| 31069 | Robertson | 0.93 | 0.33 |
| 31070 | Salisbury - Nathan | 0.81 | 0.08 |
| 31071 | Tarragindi | 0.90 | 0.23 |
| 31072 | Algester | 1.00 | 0.51 |
| 31073 | Calamvale - Stretton | 0.76 | 0.01 |
| 31074 | Pallara - Willawong | 0.98 | 0.45 |
| 31075 | Parkinson - Drewvale | 0.93 | 0.31 |
| 31076 | Rocklea - Acacia Ridge | 1.19 | 0.90 |
| 31077 | Kuraby | 0.80 | 0.08 |
| 31078 | Runcorn | 0.88 | 0.20 |
| 31079 | Sunnybank | 0.88 | 0.20 |
| 31080 | Sunnybank Hills | 0.87 | 0.12 |
| 31081 | Jindalee - Mount Ommaney | 0.90 | 0.27 |
| 31082 | Middle Park - Jamboree Heights | 0.80 | 0.09 |
| 31083 | Riverhills | 0.86 | 0.20 |
| 31084 | Seventeen Mile Rocks - Sinnamon Park | 0.78 | 0.05 |
| 31085 | Westlake | 0.77 | 0.08 |
| 31086 | Bellbowrie - Moggill | 0.85 | 0.14 |
| 31087 | Brookfield - Kenmore Hills | 0.64 | 0.00 |
| 31088 | Chapel Hill | 0.51 | 0.00 |
| 31089 | Fig Tree Pocket | 0.58 | 0.00 |
| 31090 | Kenmore | 0.56 | 0.00 |
| 31091 | Pinjarra Hills - Pullenvale | 0.75 | 0.04 |
| 31092 | Chelmer - Graceville | 0.76 | 0.06 |
| 31093 | Corinda | 0.86 | 0.19 |
| 31094 | Indooroopilly | 0.49 | 0.00 |
| 31095 | Sherwood | 0.90 | 0.26 |
| 31096 | St Lucia | 0.46 | 0.00 |
| 31097 | Taringa | 0.50 | 0.00 |
| 31098 | Enoggera | 0.70 | 0.01 |
| 31099 | Enoggera Reservoir | 0.76 | 0.09 |
| 31100 | Keperra | 1.00 | 0.50 |
| 31101 | Mitchelton | 0.76 | 0.05 |
| 31103 | The Gap | 0.73 | 0.01 |
| 31104 | Upper Kedron - Ferny Grove | 0.81 | 0.09 |
| 31105 | Brisbane City | 0.68 | 0.01 |
| 31106 | Fortitude Valley | 0.81 | 0.14 |
| 31107 | Highgate Hill | 0.94 | 0.37 |
| 31108 | Kangaroo Point | 0.73 | 0.03 |
| 31109 | New Farm | 0.79 | 0.06 |
| 31110 | South Brisbane | 0.78 | 0.07 |
| 31111 | Spring Hill | 0.73 | 0.05 |
| 31112 | West End | 0.85 | 0.18 |
| 31113 | Balmoral | 0.77 | 0.10 |
| 31114 | Bulimba | 0.61 | 0.00 |
| 31115 | East Brisbane | 0.82 | 0.13 |
| 31116 | Hawthorne | 0.74 | 0.06 |
| 31117 | Morningside - Seven Hills | 0.80 | 0.07 |
| 31118 | Norman Park | 0.88 | 0.22 |
| 31119 | Albion | 0.85 | 0.17 |
| 31120 | Alderley | 0.72 | 0.03 |
| 31121 | Ascot | 0.78 | 0.07 |
| 31122 | Clayfield | 0.72 | 0.02 |
| 31123 | Grange | 0.76 | 0.05 |
| 31124 | Hamilton (Qld) | 0.82 | 0.13 |
| 31125 | Hendra | 0.77 | 0.06 |
| 31126 | Kelvin Grove - Herston | 0.66 | 0.01 |
| 31127 | Newmarket | 0.75 | 0.05 |
| 31128 | Newstead - Bowen Hills | 0.71 | 0.01 |
| 31129 | Wilston | 0.75 | 0.06 |
| 31130 | Windsor | 0.84 | 0.15 |
| 31131 | Wooloowin - Lutwyche | 0.73 | 0.02 |
| 31132 | Ashgrove | 0.68 | 0.00 |
| 31133 | Auchenflower | 0.78 | 0.10 |
| 31134 | Bardon | 0.58 | 0.00 |
| 31135 | Paddington - Milton | 0.69 | 0.01 |
| 31136 | Red Hill (Qld) | 0.74 | 0.04 |
| 31137 | Toowong | 0.57 | 0.00 |
| 31138 | Brinsmead | 1.20 | 0.86 |
| 31139 | Clifton Beach - Kewarra Beach | 1.05 | 0.63 |
| 31140 | Freshwater - Stratford | 1.04 | 0.60 |
| 31141 | Redlynch | 0.88 | 0.19 |
| 31142 | Trinity Beach - Smithfield | 1.03 | 0.59 |
| 31143 | Yorkeys Knob - Machans Beach | 1.14 | 0.79 |
| 31144 | Bentley Park | 1.01 | 0.52 |
| 31145 | Cairns City | 1.27 | 0.96 |
| 31146 | Earlville - Bayview Heights | 1.20 | 0.90 |
| 31147 | Edmonton | 1.25 | 0.93 |
| 31148 | Gordonvale - Trinity | 1.22 | 0.92 |
| 31149 | Kanimbla - Mooroobool | 1.04 | 0.60 |
| 31151 | Manoora | 1.35 | 0.96 |
| 31152 | Manunda | 1.38 | 0.98 |
| 31153 | Mount Sheridan | 1.11 | 0.73 |
| 31154 | Westcourt - Bungalow | 1.29 | 0.97 |
| 31155 | White Rock | 1.18 | 0.84 |
| 31156 | Whitfield - Edge Hill | 1.33 | 0.98 |
| 31157 | Woree | 1.56 | 1.00 |
| 31158 | Babinda | 1.10 | 0.72 |
| 31159 | Innisfail | 1.21 | 0.90 |
| 31160 | Johnstone | 1.20 | 0.91 |
| 31161 | Tully | 1.20 | 0.92 |
| 31163 | Yarrabah | 1.37 | 0.92 |
| 31164 | Daintree | 1.35 | 0.98 |
| 31165 | Port Douglas | 1.08 | 0.69 |
| 31166 | Atherton | 0.91 | 0.24 |
| 31167 | Herberton | 1.20 | 0.91 |
| 31168 | Kuranda | 1.09 | 0.71 |
| 31169 | Malanda - Yungaburra | 1.04 | 0.63 |
| 31170 | Mareeba | 1.09 | 0.74 |
| 31171 | Balonne | 1.04 | 0.59 |
| 31172 | Chinchilla | 0.88 | 0.19 |
| 31173 | Goondiwindi | 1.04 | 0.57 |
| 31174 | Inglewood - Waggamba | 0.97 | 0.42 |
| 31175 | Miles - Wandoan | 0.89 | 0.24 |
| 31176 | Roma | 0.87 | 0.25 |
| 31177 | Roma Region | 0.99 | 0.46 |
| 31178 | Tara | 1.04 | 0.59 |
| 31179 | Crows Nest - Rosalie | 0.90 | 0.21 |
| 31180 | Jondaryan | 0.90 | 0.24 |
| 31181 | Millmerran | 0.95 | 0.38 |
| 31182 | Pittsworth | 0.78 | 0.05 |
| 31183 | Wambo | 0.81 | 0.04 |
| 31184 | Clifton - Greenmount | 0.74 | 0.03 |
| 31185 | Southern Downs - East | 0.82 | 0.12 |
| 31186 | Southern Downs - West | 0.85 | 0.13 |
| 31187 | Stanthorpe | 1.06 | 0.63 |
| 31188 | Stanthorpe Region | 0.75 | 0.04 |
| 31189 | Warwick | 0.85 | 0.11 |
| 31190 | Central Highlands - East | 1.05 | 0.62 |
| 31191 | Central Highlands - West | 1.11 | 0.78 |
| 31192 | Emerald | 1.31 | 0.93 |
| 31205 | Berserker | 1.11 | 0.76 |
| 31206 | Bouldercombe | 1.06 | 0.64 |
| 31207 | Emu Park | 1.34 | 0.97 |
| 31208 | Frenchville - Mount Archer | 1.10 | 0.74 |
| 31209 | Glenlee - Rockyview | 0.96 | 0.41 |
| 31210 | Gracemere | 1.14 | 0.81 |
| 31211 | Lakes Creek | 1.35 | 0.96 |
| 31212 | Mount Morgan | 1.26 | 0.91 |
| 31213 | Norman Gardens | 0.88 | 0.18 |
| 31214 | Park Avenue | 1.02 | 0.56 |
| 31215 | Parkhurst - Kawana | 0.96 | 0.39 |
| 31216 | Rockhampton - West | 1.12 | 0.77 |
| 31217 | Rockhampton City | 1.31 | 0.96 |
| 31218 | Rockhampton Region - East | 1.11 | 0.75 |
| 31219 | Rockhampton Region - North | 0.98 | 0.44 |
| 31220 | Rockhampton Region - West | 1.09 | 0.70 |
| 31221 | Shoalwater Bay | 1.02 | 0.53 |
| 31222 | The Range - Allenstown | 1.26 | 0.93 |
| 31223 | Yeppoon | 1.06 | 0.69 |
| 31224 | Broadbeach Waters | 1.01 | 0.52 |
| 31225 | Burleigh Heads | 0.89 | 0.18 |
| 31226 | Burleigh Waters | 0.91 | 0.22 |
| 31227 | Mermaid Beach - Broadbeach | 0.89 | 0.19 |
| 31228 | Mermaid Waters | 0.92 | 0.26 |
| 31229 | Miami | 0.92 | 0.30 |
| 31230 | Coolangatta | 0.56 | 0.00 |
| 31231 | Currumbin - Tugun | 0.72 | 0.01 |
| 31232 | Currumbin Waters | 0.97 | 0.42 |
| 31233 | Elanora | 0.89 | 0.19 |
| 31234 | Palm Beach | 1.04 | 0.61 |
| 31235 | Arundel | 0.98 | 0.45 |
| 31236 | Biggera Waters | 1.16 | 0.85 |
| 31237 | Coombabah | 1.11 | 0.81 |
| 31238 | Labrador | 1.31 | 1.00 |
| 31239 | Paradise Point - Hollywell | 0.96 | 0.38 |
| 31240 | Runaway Bay | 0.88 | 0.17 |
| 31241 | Guanaba - Springbrook | 0.94 | 0.35 |
| 31242 | Tamborine - Canungra | 0.96 | 0.35 |
| 31243 | Currumbin Valley - Tallebudgera | 0.97 | 0.43 |
| 31244 | Mudgeeraba - Bonogin | 0.86 | 0.11 |
| 31245 | Reedy Creek - Andrews | 0.99 | 0.48 |
| 31246 | Carrara | 1.03 | 0.59 |
| 31247 | Highland Park | 1.01 | 0.53 |
| 31248 | Nerang - Mount Nathan | 1.22 | 0.97 |
| 31249 | Pacific Pines - Gaven | 1.07 | 0.70 |
| 31250 | Worongary - Tallai | 0.83 | 0.10 |
| 31251 | Coomera | 1.21 | 0.89 |
| 31252 | Helensvale | 0.82 | 0.05 |
| 31253 | Hope Island | 0.92 | 0.26 |
| 31254 | Jacobs Well - Alberton | 1.17 | 0.83 |
| 31255 | Ormeau - Yatala | 0.86 | 0.13 |
| 31256 | Oxenford - Maudsland | 0.92 | 0.26 |
| 31257 | Pimpama | 1.20 | 0.87 |
| 31258 | Upper Coomera - Willow Vale | 1.02 | 0.56 |
| 31259 | Clear Island Waters | 0.93 | 0.31 |
| 31260 | Merrimac | 0.99 | 0.46 |
| 31261 | Robina | 0.82 | 0.03 |
| 31262 | Varsity Lakes | 0.95 | 0.36 |
| 31263 | Ashmore | 1.00 | 0.49 |
| 31264 | Molendinar | 1.06 | 0.65 |
| 31265 | Parkwood | 1.07 | 0.67 |
| 31267 | Benowa | 0.90 | 0.23 |
| 31268 | Bundall | 1.06 | 0.65 |
| 31269 | Main Beach | 0.66 | 0.02 |
| 31270 | Surfers Paradise | 0.97 | 0.40 |
| 31271 | Darra - Sumner | 0.91 | 0.28 |
| 31272 | Durack | 1.01 | 0.54 |
| 31273 | Forest Lake - Doolandella | 0.93 | 0.28 |
| 31274 | Inala - Richlands | 1.54 | 1.00 |
| 31275 | Oxley (Qld) | 1.08 | 0.70 |
| 31276 | Wacol | 1.41 | 0.98 |
| 31277 | Boonah | 0.80 | 0.04 |
| 31278 | Esk | 1.00 | 0.51 |
| 31280 | Lockyer Valley - East | 0.95 | 0.32 |
| 31281 | Lowood | 1.02 | 0.58 |
| 31282 | Rosewood | 0.91 | 0.23 |
| 31283 | Brassall | 1.16 | 0.86 |
| 31284 | Bundamba | 1.06 | 0.65 |
| 31285 | Churchill - Yamanto | 1.07 | 0.65 |
| 31286 | Ipswich - Central | 1.23 | 0.92 |
| 31287 | Ipswich - East | 1.11 | 0.82 |
| 31288 | Ipswich - North | 0.94 | 0.35 |
| 31289 | Karalee - Barellan Point | 0.93 | 0.33 |
| 31290 | Karana Downs | 0.86 | 0.17 |
| 31291 | Leichhardt - One Mile | 1.18 | 0.86 |
| 31292 | North Ipswich - Tivoli | 1.13 | 0.80 |
| 31293 | Raceview | 0.87 | 0.16 |
| 31294 | Ripley | 0.90 | 0.27 |
| 31295 | Riverview | 0.95 | 0.39 |
| 31296 | Bellbird Park - Brookwater | 1.01 | 0.51 |
| 31297 | Camira - Gailes | 1.14 | 0.81 |
| 31298 | Carole Park |  |  |
| 31299 | Collingwood Park - Redbank | 1.19 | 0.87 |
| 31300 | Goodna | 1.05 | 0.62 |
| 31302 | Redbank Plains | 1.12 | 0.79 |
| 31303 | Springfield | 0.91 | 0.31 |
| 31304 | Springfield Lakes | 0.92 | 0.30 |
| 31305 | Beaudesert | 0.91 | 0.24 |
| 31306 | Beenleigh | 1.08 | 0.70 |
| 31307 | Eagleby | 1.06 | 0.68 |
| 31308 | Edens Landing - Holmview | 1.10 | 0.71 |
| 31309 | Mount Warren Park | 1.02 | 0.55 |
| 31310 | Wolffdene - Bahrs Scrub | 1.02 | 0.56 |
| 31311 | Boronia Heights - Park Ridge | 1.20 | 0.93 |
| 31312 | Browns Plains | 1.04 | 0.61 |
| 31313 | Chambers Flat - Logan Reserve | 1.03 | 0.58 |
| 31314 | Crestmead | 1.32 | 0.97 |
| 31316 | Hillcrest | 1.07 | 0.68 |
| 31317 | Marsden | 1.19 | 0.88 |
| 31318 | Munruben - Park Ridge South | 0.94 | 0.36 |
| 31319 | Regents Park - Heritage Park | 1.21 | 0.92 |
| 31320 | Greenbank | 0.99 | 0.47 |
| 31321 | Jimboomba | 1.16 | 0.90 |
| 31322 | Logan Village | 1.02 | 0.55 |
| 31323 | Bethania - Waterford | 1.18 | 0.91 |
| 31324 | Cornubia - Carbrook | 0.87 | 0.17 |
| 31325 | Loganholme - Tanah Merah | 0.89 | 0.20 |
| 31326 | Loganlea | 1.09 | 0.72 |
| 31327 | Shailer Park | 0.73 | 0.01 |
| 31328 | Waterford West | 1.15 | 0.82 |
| 31329 | Daisy Hill | 0.93 | 0.31 |
| 31330 | Kingston (Qld.) | 1.57 | 1.00 |
| 31331 | Logan Central | 1.07 | 0.66 |
| 31332 | Rochedale South - Priestdale | 1.05 | 0.64 |
| 31333 | Slacks Creek | 1.20 | 0.91 |
| 31334 | Springwood | 0.92 | 0.29 |
| 31335 | Underwood | 1.07 | 0.66 |
| 31336 | Woodridge | 1.15 | 0.84 |
| 31337 | Bowen | 1.29 | 0.95 |
| 31338 | Broadsound - Nebo | 1.13 | 0.79 |
| 31339 | Clermont | 0.91 | 0.29 |
| 31340 | Collinsville | 1.15 | 0.82 |
| 31341 | Moranbah | 1.02 | 0.54 |
| 31342 | Andergrove - Beaconsfield | 1.03 | 0.59 |
| 31343 | East Mackay | 1.10 | 0.69 |
| 31344 | Eimeo - Rural View | 0.99 | 0.47 |
| 31345 | Eungella Hinterland | 1.06 | 0.62 |
| 31346 | Mackay | 1.16 | 0.78 |
| 31347 | Mackay Harbour | 0.95 | 0.41 |
| 31348 | Mount Pleasant - Glenella | 0.83 | 0.09 |
| 31349 | North Mackay | 1.07 | 0.66 |
| 31350 | Ooralea - Bakers Creek | 0.99 | 0.48 |
| 31351 | Pioneer Valley | 1.11 | 0.75 |
| 31352 | Sarina | 1.08 | 0.71 |
| 31353 | Seaforth - Calen | 1.00 | 0.50 |
| 31354 | Shoal Point - Bucasia | 1.01 | 0.51 |
| 31355 | Slade Point | 0.97 | 0.44 |
| 31356 | South Mackay | 1.11 | 0.74 |
| 31357 | Walkerston - Eton | 1.02 | 0.56 |
| 31358 | West Mackay | 1.04 | 0.60 |
| 31359 | Airlie - Whitsundays | 1.16 | 0.81 |
| 31361 | Proserpine | 1.04 | 0.60 |
| 31362 | Beachmere - Sandstone Point | 1.37 | 1.00 |
| 31363 | Bribie Island | 1.27 | 1.00 |
| 31364 | Burpengary - East | 1.17 | 0.83 |
| 31365 | Caboolture | 1.36 | 1.00 |
| 31366 | Caboolture - South | 1.41 | 1.00 |
| 31367 | Elimbah | 1.21 | 0.87 |
| 31368 | Morayfield - East | 1.08 | 0.69 |
| 31369 | Wamuran | 1.14 | 0.79 |
| 31370 | Kilcoy | 0.91 | 0.25 |
| 31371 | Woodford - D'Aguilar | 1.06 | 0.66 |
| 31372 | Burpengary | 1.11 | 0.80 |
| 31373 | Deception Bay | 1.17 | 0.94 |
| 31374 | Morayfield | 1.07 | 0.66 |
| 31375 | Narangba | 0.98 | 0.44 |
| 31376 | Upper Caboolture | 1.08 | 0.67 |
| 31377 | Clontarf | 1.38 | 0.99 |
| 31378 | Margate - Woody Point | 1.54 | 1.00 |
| 31379 | Redcliffe | 1.18 | 0.90 |
| 31380 | Rothwell - Kippa-Ring | 1.24 | 0.98 |
| 31382 | Albany Creek | 0.89 | 0.17 |
| 31383 | Cashmere | 0.93 | 0.30 |
| 31384 | Dayboro | 0.99 | 0.48 |
| 31385 | Eatons Hill | 0.87 | 0.18 |
| 31386 | The Hills District | 0.71 | 0.00 |
| 31387 | Samford Valley | 0.75 | 0.02 |
| 31388 | Dakabin - Kallangur | 1.22 | 0.97 |
| 31389 | Murrumba Downs - Griffin | 0.87 | 0.15 |
| 31390 | North Lakes - Mango Hill | 0.93 | 0.28 |
| 31391 | Bray Park | 1.10 | 0.75 |
| 31392 | Lawnton | 1.27 | 0.95 |
| 31393 | Petrie | 0.93 | 0.33 |
| 31394 | Strathpine - Brendale | 1.13 | 0.82 |
| 31395 | Aurukun | 1.30 | 0.86 |
| 31396 | Cape York | 1.29 | 0.95 |
| 31397 | Croydon - Etheridge | 1.16 | 0.79 |
| 31398 | Kowanyama - Pormpuraaw | 1.38 | 0.93 |
| 31399 | Northern Peninsula | 1.29 | 0.86 |
| 31400 | Tablelands | 1.18 | 0.89 |
| 31401 | Torres | 1.21 | 0.81 |
| 31402 | Torres Strait Islands | 1.16 | 0.70 |
| 31403 | Weipa | 1.20 | 0.76 |
| 31404 | Carpentaria | 1.17 | 0.82 |
| 31405 | Mount Isa | 1.18 | 0.84 |
| 31406 | Mount Isa Region | 1.12 | 0.73 |
| 31407 | Northern Highlands | 1.05 | 0.62 |
| 31408 | Barcaldine - Blackall | 1.02 | 0.56 |
| 31409 | Charleville | 1.03 | 0.56 |
| 31410 | Far Central West | 1.13 | 0.75 |
| 31411 | Far South West | 1.15 | 0.80 |
| 31412 | Longreach | 1.13 | 0.75 |
| 31413 | Buderim - North | 0.78 | 0.01 |
| 31414 | Buderim - South | 0.77 | 0.02 |
| 31415 | Mountain Creek | 0.72 | 0.02 |
| 31416 | Sippy Downs | 0.80 | 0.06 |
| 31417 | Aroona - Currimundi | 0.96 | 0.38 |
| 31418 | Buddina - Minyama | 0.83 | 0.12 |
| 31419 | Caloundra - Kings Beach | 0.99 | 0.46 |
| 31420 | Caloundra - West | 1.05 | 0.67 |
| 31421 | Golden Beach - Pelican Waters | 0.87 | 0.12 |
| 31422 | Moffat Beach - Battery Hill | 0.95 | 0.37 |
| 31423 | Parrearra - Warana | 0.88 | 0.17 |
| 31424 | Wurtulla - Birtinya | 0.94 | 0.33 |
| 31425 | Coolum Beach | 0.86 | 0.12 |
| 31426 | Marcoola - Mudjimba | 0.93 | 0.29 |
| 31427 | Maroochydore - Kuluin | 1.04 | 0.66 |
| 31428 | Mooloolaba - Alexandra Headland | 0.84 | 0.08 |
| 31434 | Noosa Heads | 0.75 | 0.04 |
| 31435 | Noosaville | 0.74 | 0.01 |
| 31437 | Sunshine Beach | 0.73 | 0.03 |
| 31438 | Tewantin | 1.05 | 0.64 |
| 31439 | Beerwah | 1.06 | 0.67 |
| 31440 | Caloundra Hinterland | 0.75 | 0.01 |
| 31441 | Glass House Mountains | 1.04 | 0.60 |
| 31442 | Landsborough | 0.89 | 0.20 |
| 31443 | Maroochy Hinterland | 0.91 | 0.24 |
| 31444 | Palmwoods | 0.85 | 0.10 |
| 31445 | Cambooya - Wyreema | 0.64 | 0.00 |
| 31446 | Darling Heights | 0.59 | 0.00 |
| 31447 | Drayton - Harristown | 0.78 | 0.04 |
| 31448 | Gatton | 0.97 | 0.42 |
| 31449 | Gowrie (Qld) | 0.84 | 0.14 |
| 31450 | Highfields | 0.62 | 0.00 |
| 31451 | Lockyer Valley - West | 0.77 | 0.02 |
| 31452 | Middle Ridge | 0.64 | 0.00 |
| 31453 | Newtown (Qld) | 0.69 | 0.01 |
| 31454 | North Toowoomba - Harlaxton | 0.69 | 0.01 |
| 31455 | Rangeville | 0.64 | 0.00 |
| 31456 | Toowoomba - Central | 0.79 | 0.04 |
| 31457 | Toowoomba - East | 0.62 | 0.00 |
| 31458 | Toowoomba - West | 0.67 | 0.00 |
| 31459 | Wilsonton | 0.87 | 0.13 |
| 31460 | Ayr | 1.16 | 0.84 |
| 31461 | Burdekin | 1.15 | 0.84 |
| 31462 | Charters Towers | 1.18 | 0.85 |
| 31463 | Dalrymple | 1.16 | 0.85 |
| 31464 | Ingham | 1.25 | 0.89 |
| 31465 | Ingham Region | 0.91 | 0.26 |
| 31466 | Palm Island | 1.46 | 0.90 |
| 31467 | Aitkenvale | 1.04 | 0.61 |
| 31468 | Annandale | 1.02 | 0.55 |
| 31469 | Belgian Gardens - Pallarenda | 1.09 | 0.70 |
| 31470 | Bohle Plains | 1.18 | 0.85 |
| 31471 | Condon - Rasmussen | 1.18 | 0.89 |
| 31472 | Cranbrook | 1.10 | 0.75 |
| 31473 | Deeragun | 1.39 | 0.99 |
| 31474 | Douglas | 1.02 | 0.54 |
| 31475 | Garbutt - West End | 1.23 | 0.92 |
| 31476 | Gulliver - Currajong - Vincent | 1.19 | 0.88 |
| 31477 | Heatley | 1.17 | 0.85 |
| 31478 | Hermit Park - Rosslea | 1.52 | 1.00 |
| 31479 | Hyde Park - Pimlico | 1.60 | 1.00 |
| 31480 | Kelso | 1.15 | 0.82 |
| 31481 | Kirwan - East | 1.09 | 0.71 |
| 31482 | Kirwan - West | 1.10 | 0.75 |
| 31483 | Magnetic Island | 1.12 | 0.70 |
| 31484 | Mount Louisa | 1.16 | 0.84 |
| 31485 | Mundingburra | 1.13 | 0.78 |
| 31486 | Northern Beaches | 1.35 | 0.98 |
| 31487 | Oonoonba | 1.01 | 0.52 |
| 31488 | South Townsville - Railway Estate | 1.20 | 0.87 |
| 31489 | Townsville - South | 1.20 | 0.88 |
| 31490 | Townsville City - North Ward | 1.06 | 0.64 |
| 31491 | Wulguru - Roseneath | 1.20 | 0.88 |
| 31492 | Ashfield - Kepnock | 1.13 | 0.79 |
| 31493 | Bargara - Burnett Heads | 1.07 | 0.73 |
| 31494 | Branyan - Kensington | 1.09 | 0.70 |
| 31495 | Bundaberg | 1.45 | 0.99 |
| 31496 | Bundaberg East - Kalkie | 1.37 | 0.98 |
| 31497 | Bundaberg North - Gooburrum | 1.12 | 0.81 |
| 31498 | Bundaberg Region - North | 1.10 | 0.78 |
| 31499 | Bundaberg Region - South | 1.32 | 0.99 |
| 31500 | Millbank - Avoca | 1.08 | 0.72 |
| 31501 | Svensson Heights - Norville | 1.20 | 0.89 |
| 31502 | Walkervale - Avenell Heights | 1.15 | 0.87 |
| 31503 | Gayndah - Mundubbera | 1.06 | 0.66 |
| 31504 | Gin Gin | 1.18 | 0.88 |
| 31505 | Kingaroy | 1.00 | 0.49 |
| 31506 | Kingaroy Region - North | 1.14 | 0.85 |
| 31507 | Kingaroy Region - South | 0.85 | 0.16 |
| 31508 | Monto - Eidsvold | 1.01 | 0.52 |
| 31509 | Nanango | 1.06 | 0.68 |
| 31510 | North Burnett | 1.11 | 0.69 |
| 31511 | Cooloola | 1.23 | 0.94 |
| 31512 | Gympie - North | 1.22 | 0.95 |
| 31513 | Gympie - South | 0.85 | 0.17 |
| 31514 | Gympie Region | 1.04 | 0.63 |
| 31515 | Kilkivan | 1.14 | 0.81 |
| 31516 | Booral - River Heads | 1.20 | 0.88 |
| 31517 | Craignish - Dundowran Beach | 1.23 | 0.90 |
| 31518 | Pialba - Eli Waters | 0.96 | 0.37 |
| 31519 | Point Vernon | 1.58 | 1.00 |
| 31520 | Torquay - Scarness - Kawungan | 1.19 | 0.95 |
| 31521 | Urangan - Wondunna | 1.07 | 0.72 |
| 31522 | Burrum - Fraser | 1.23 | 0.96 |
| 31523 | Granville | 1.00 | 0.50 |
| 31524 | Maryborough (Qld) | 1.17 | 0.93 |
| 31525 | Maryborough Region - South | 1.12 | 0.82 |
| 31526 | Tinana | 1.11 | 0.76 |
| 31527 | Redland Islands | 1.45 | 1.00 |
| 31528 | Banana | 0.82 | 0.07 |
| 31529 | Biloela | 1.07 | 0.62 |
| 31530 | Agnes Water - Miriam Vale | 1.32 | 0.97 |
| 31531 | Boyne Island - Tannum Sands | 1.13 | 0.77 |
| 31532 | Callemondah | 1.10 | 0.67 |
| 31533 | Clinton - New Auckland | 1.09 | 0.71 |
| 31534 | Gladstone | 1.28 | 0.93 |
| 31535 | Gladstone Hinterland | 1.03 | 0.57 |
| 31536 | Kin Kora - Sun Valley | 1.06 | 0.62 |
| 31538 | Telina - Toolooa | 1.00 | 0.49 |
| 31539 | West Gladstone | 1.11 | 0.72 |
| 31540 | Southport - North | 1.25 | 0.97 |
| 31541 | Southport - South | 1.08 | 0.75 |
| 31542 | Scarborough - Newport - Moreton Island | 0.94 | 0.31 |
| 31543 | Peregian Beach - Marcus Beach | 0.77 | 0.06 |
| 31544 | Peregian Springs | 0.83 | 0.11 |
| 31545 | Bli Bli | 0.98 | 0.45 |
| 31546 | Diddillibah - Rosemount | 0.94 | 0.36 |
| 31547 | Eumundi - Yandina | 0.93 | 0.30 |
| 31548 | Nambour | 1.16 | 0.92 |
| 31549 | Noosa Hinterland | 0.83 | 0.04 |

# Appendix 3 List of area-level variables

| Sources | N | Variables |
| --- | --- | --- |
| ABS | 171 | Agriculture Forestry and Fishing; Mining; Manufacturing; Electricity gas water and waste; Construction; Wholesale trade; Retail trade; Accommodation and food; Transport postal and warehousing; Information media and telecommunications; Financial and insurance; Rental hiring and real estate; Professional scientific and technical; Administrative and support; Public administration and safety; Education and training; Health care and social assistance; Arts and recreation; Other; Inadequately described;  People 15-64; Males 0-4; Males 5-9; Males 10-14; Males 15-19; Males 20-24; Males 25-29; Males 30-34; Males 35-39; Males 40-44; Males 45-49; Males 50-54; Males 55-59; Males 60-64; Males 65-69; Males 70-74; Males 75-79; Males 80-84; Males 85+; Females 0-4; Females 5-9; Females 10-14; Females 15-19; Females 20-24; Females 25-29; Females 30-34; Females 35-39; Females 40-44; Females 45-49; Females 50-54; Females 55-59; Females 60-64; Females 65-69; Females 70-74; Females 75-79; Females 80-84; Females 85+; Aboriginal Torres Strait Islander peoples; Born in Oceania excluding Australia; Born in North West Europe; Born in Southern Eastern Europe; Born in South East Asia; Born in North East Asia; Born Southern Central Asia; Born in Americas; Born in Sub Saharan Africa; Total born overseas; Buddhism; Christianity; Hinduism; Other religions; Other spiritual beliefs; No religion secular beliefs; Inadequately described stated; Australian citizen; Not an Australian citizen; Australian citizenship not stated; Speaks other language at home.  Median employee income per year; Employee income as main source; Median own unincorporated business income per year; Own unincorporated business income as main source; Median investment income per year; Investment income as main source; Median Super income per year; Super income as main source; Median total income per year; Median Gross Capital Gains; Persons earning 1-499 per week; Persons earning 500-999 per week; Persons earning 1000-1999 per week; Persons earning 2000-2999 per week; Persons earning over 3000 per week; Persons earning nil income; Persons earning negative income; persons income not stated; Median total household income per week;  Persons with a disability; Persons with severe core activity limitation; Persons with moderate or mild core activity limitation; Persons who are primary carers; Persons who are nonprimary carers; Persons need assistance with core activities; private insurance rate per 1000;  Average household size; Average family size; Married percentage; Never married percentage; Widowed percentage; Divorced percentage; Separated percentage; Internet accessed; Internet not accessed; Average monthly household rental payment; Average monthly household mortgage payment; Owned dwellings outright percentage; Owned dwellings with a mortgage; Rented dwellings percentage; Mortgage payment less than 30 percent of income; Mortgage payment more than 30 percent of income; Rental payment less than 30 percent of income; Rental payment more than 30 percent of income; IRSD Aus rank; IRSAD Aus rank; IEO Aus rank; IER Aus rank; IRSD state rank; IRSAD state rank; IEO state rank; IER state rank; IHAD Q1; IHAD Q2; IHAD Q3; IHAD Q4; Homeless rate per 10000;  Completed year 12; Completed year 11; Completed year 10; Completed year 9; Completed year 8 or below; Did not go to school; Has school qualifications; Postgraduate degree; Graduate Diploma or Graduate Certificate; Bachelor degree; Advanced diploma or diploma; Certificate; study field natural and physical science; study field IT; study field Engineering; study field Building; Study field agriculture environmental; Study field health; Study field education; Study field management and commerce; study field society and culture; study field creative arts; study field food services; Study field mixed field; work part study fulltime; work fulltime; study fulltime; Unemployed rate; Managers; Professionals; Technicians and trades workers; Community and personal service workers; Clerical and administrative workers; Sales workers; Machinery operators and drivers; Labourers. |
| PHIDU | 12 | High blood pressure; Overweight; Obesity; Smoking; Adequate fruit intake; Low exercise; Diabetes mellitus; Mental and behaviour problems; heart, stroke and vascular disease; Asthma; Chronic obstructive pulmonary disease; Osteoporosis. |
| NHSD | 13 | General practitioner; Pathology facilities; Medical imaging services; Breast cancer treatment facilities; Other general hospital and emergency services; Women and children’s health; General community health services; Social and mental support services; Healthy lifestyle services; Other cancer services; Other disease management; Aged care services; Other. |

# Appendix 4 Categories of health services in Queensland

| Type of services | Name of services |
| --- | --- |
| 1. General practitioner | General practice service; General medical service; Walk-in centre; Aboriginal and Torres Strait Islander health service; Refugee health service |
| 2. Pathology facilities | Clinical pathology service; Genetic pathology service; Chemical pathology service |
| 3. Medical imaging services | Nuclear medicine service; Ultrasound service; Magnetic resonance imaging service; Radiology service; Breast screening service^1^ |
| 4. Breast cancer treatment facilities | Chemotherapy service; Surgical oncology service; Clinical oncology service; Radiation oncology service; Private same-day hospital service; Hospital-based outpatient clinic; Hospital service |
| 5. Other general hospital and emergency services | General surgical service; Anaesthetic service; Hospital pharmacy service; Clinical pharmacology service; Emergency department service; Air ambulance service; Patient transport service |
| 6. Women and children’s health | Women's health service; Maternal, child and family health service; Obstetric ultrasound service; Obstetrics and gynaecology service; Perinatology service; Gynaecological oncology service; Reproductive endocrinology and infertility service; Family planning service; Perinatal mental health service; Obstetrics service; Gynaecology service; Early parenting support service; Parenting and family support service; Urogynaecology service; Midwifery service; Neonatology service; Paediatric cardiology service; Paediatric clinical genetics service; Paediatric clinical pharmacology service; Paediatric dentistry service; Paediatric dermatology service; Paediatric endocrinology service; Paediatric gastroenterology and hepatology service; Paediatric haematology service; Paediatric immunology and allergy service; Paediatric infectious disease service; Paediatric intensive care service; Paediatric medical service; Paediatric nephrology service; Paediatric neurology service; Paediatric nuclear medicine service; Paediatric oncology service; Paediatric otolaryngology service; Paediatric rehabilitation medicine service; Paediatric respiratory service; Paediatric rheumatology service; Paediatric sleep medicine service; Paediatric surgical service; Paediatric thoracic medicine service |
| 7. General community health services | Immunisation service; Medication management service; Pharmacy service; Community health service; Community health services; Nursing service; Health information and referral service; Health counselling service; Nurse led clinic; Nurse practitioner led clinic; |
| 8. Social and mental support services | Kinship care service; Friendly visiting service; Psychiatry service; Psychology service; Mental health information and referral service; Generalist counselling service; Support group service; Patient advocacy and liaison service; Clinical psychology service; Mental health service; Adult mental health service; Health promotion service; Mental health case management service; Mental health advocacy service; Mental health crisis assessment and treatment service; One on one mentoring support service; Crisis counselling; Social work service; Acute mental health inpatient service; Child and adolescent mental health service; Family counselling service; Financial counselling service; Food relief service; Foster care service; Home care support service; Integrated Family Service; Intensive care service; Leaving care support service; Mediation service; Mental health non-residential rehabilitation service; Mental health residential rehabilitation service; Palliative medicine service; Relationship counselling service; Respite care service; Social support service; Accommodation support service; Bereavement counselling service; Community transport service; Crisis and emergency accommodation service; Delivered meal service; |
| 9. Healthy lifestyle services | Alcohol and drug information service; Drug and alcohol counselling service; Drug and alcohol support group service; Physical activity program; Physical and rehabilitation medicine service; Pharmacotherapy service for drug and alcohol dependence; Non-residential drug and alcohol dependence treatment service; Problem gambling counselling service; Residential drug and alcohol dependence treatment service; Smoking cessation service; Addiction medicine service; Dietetics service; Exercise physiology service; Nutrition service |
| 10. Other cancer services | Cancer support group service; Carer support service; Community cancer service^2^; Skin cancer clinic; General practice skin cancer service |
| 11. Other disease management^3^ | Chronic disease management service; Cardiac rehabilitation service; Chiropractic service; Diabetic education service; Endocrinology service; Gastroenterology and hepatology service; Gastroenterology service; Haematology service; Hepatology service; Immunology service; Allergy and clinical immunology service; Nephrology service; Neurology service; Neuropsychology service; Occupational medicine service; Occupational therapy service; Pain management service; Pain medicine service; Physiotherapy service; Podiatry service; Renal dialysis service; Respiratory medicine service; Rheumatology service; Sleep medicine service; Thoracic medicine service; Sports medicine service; Cardiology service; Cardiothoracic surgery service; Colorectal surgery service; Continence Service; Dermatological surgery service; Dermatology service; Hand surgery service; Neurosurgical service; Vascular surgery service; Acquired brain injury information and referral support service; Audiological service; Clinical genetics service; Endoscopy service; Genetic counselling service; Infectious diseases service; Men's health clinic service; Plastic surgery service; Prosthetics and orthotics service; Trauma counselling service; Urology service; Wound management service; Medical microbiology service; |
| 12. Aged care services | Aged care; Aged care assessment service; Aged care information and referral service; Aged care planned activity group; Aged care residential respite high care; Aged care residential respite low care;  Aged care transport service; Geriatric medicine service; Permanent residential aged care service; Personal care for older persons service; Aged care case management service |
| 13. Other^4^ | Acupuncture service; Child protection service; Child residential care service; Chinese herbal medicine service; Community youth support service; Dental hygiene service; Disability advocacy service; Disability aids and equipment service; Disability case management service; Disability financial support service; Disability information and referral service; Disability social and recreational activity service; Disability supported accommodation service; Disability transport service; Domestic and family violence support service; Domestic violence counselling service; Early childhood intervention service; Endodontic service; Garden maintenance service; General dental practice service; Hearing aid service; Home maintenance and repair service; Home modification service; Homelessness support service; Hydrotherapy service; Massage therapy service; Music therapy service; Myotherapy service; Needle and Syringe Program; Ophthalmology service; Optometry service; Oral and axillofacial surgery; Oral medicine service; Oral surgery service; Orthodontics service; Orthopaedic service; Osteopathy service; Otolaryngology service; Outreach service; Paediatric rheumatology service; Periodontic service; Personal alarm service; Prosthodontic service; Public health medicine; Sexual assault counselling service; Sexual health service; Speech and language therapy service; Spiritual counselling service; Victims of crime counselling service |

1. Breast screening facilities are not complete based on Breastscreen website [11]; Mobile screening facilities in remote area were not included.

2. This is a mixture of skin cancer treatment/cancer scanning/cancer consultant

3. Ophthalmology and Dentistry services were not included.

4. All the other NHSD facilities.

# Appendix 5 Number of health facilities within each category in Queensland

| **Type** | **Freq.** | **Percent** |
| --- | --- | --- |
| General practitioner | 1,986 | 8.04 |
| Pathology facilities | 170 | 0.69 |
| Medical imaging services | 272 | 1.10 |
| Breast cancer treatment facilities | 421 | 1.70 |
| Other general hospital and emergency services | 523 | 2.12 |
| Women and children's health services | 1,210 | 4.90 |
| General community health services | 2,972 | 12.04 |
| Social and mental support services | 4,864 | 19.70 |
| Healthy lifestyle services | 1,745 | 7.07 |
| Other cancer services | 201 | 0.81 |
| Other disease management | 3,866 | 15.66 |
| Aged care services | 2,421 | 9.80 |
| Other | 4,042 | 16.37 |
| **Total** | **24,693** | **100** |

# Appendix 6 The density of health services in each SA2 area

We classified 194 different types of health-related services into 13 categories (Appendix 4). In Queensland, as of November 2020, a total of 24,693 facilities fell within these 13 categories. For each category, the longitude and latitude of all the relevant health facilities were mapped in Queensland. A buffer zone was established around each health facility, defined as a 10-kilometer travel distance by road, capturing the geographical reach of the facility while considering actual travel routes. The density of each health services category within each SA2 area was determined using the formula:

$$D_{a}=\frac{N_{a}}{A_{a}}$$

Where

$D_{a}$ is the density of health services in SA2 area a;

$N_{a}$ is the number of health facilities with buffer zones intersecting with SA2 area a;

$A_{a}$ is the area of SA2 area a in square kilometres.

# Appendix 7 Introduction of model evaluation statistics

The performances of the final models were evaluated using a confusion matrix based on the test set, which accounted for 20% of the entire dataset (total number of areas = 103). A confusion matrix (Table A6) is a structured table that enables visualization of a model's performance by showing actual versus predicted values across categories [12]. In Table A6, 'A' represents true positives (TP), where both the actual and predicted values are positive; 'D' represents true negatives (TN), where both the actual and predicted values are negative; 'B' represents false positives (FP), where the predicted value is positive but the actual value is negative; and 'C' represents false negatives (FN), where the predicted value is negative but the actual value is positive.

Table A6 An example of confusion matrix.

| Predicted | Actual | |
| --- | --- | --- |
|  | Positive | Negative |
| Positive | A | B |
| Negative | C | D |

From this confusion matrix, we calculated accuracy and the Kappa statistic (as described below) to evaluate model performance. The model with the highest accuracy and Kappa was selected as the final model.

Accuracy represents the proportion of observations that were correctly predicted and is calculated as follows:

$$Accuracy=\frac{A+B}{A+B+C+D}$$

The 95% confidence interval (95% CI) of accuracy was calculated using the Wilson score interval to provide an accurate CI for proportions [13]. The accuracy can be compared with the no information rate (NIR), which is used to evaluate a model's performance against a simple strategy where it would always predict the most frequent class.

$$NIR=\max\left( A+C, B+ D \right)/(A+B+C+D)$$

Kappa measures model performance by accounting for the agreement occurring by chance [14]. It is calculated as:

$$Kappa=\frac{Observed agreement-Expected aggreement}{1-Expected agreement}$$

Where the observed agreement is accuracy. The Expected Agreement represents the level of agreement that could happen by chance, based on the proportions of each class in the actual and predicted data, which is calculated as:

$$Expected aggreement=\frac{\left( A+B \right)\times\left( A+C \right)+(C+D)\times(B+D)}{A+B+C+D}$$

In addition, for each of the three lung cancer incidence categories, we also reported sensitivity and specificity, which were also calculated based on the confusion matrix [15].

Sensitivity (or Recall) measures the model’s ability to correctly identify positive instances. It is calculated as:

$$Sensitivity=\frac{A}{A+C}$$

Specificity measures the model's ability to correctly identify negative instances. It is calculated as:

$$Specificity=\frac{D}{B+D}$$

# Appendix 8 Example of gap adjusted percentile ranking.

| Observation | Feature value | Rank | Percentile rank | Gap adjusted rank | Gap adjusted percentile rank |
| --- | --- | --- | --- | --- | --- |
| 1 | 1 | 1 | 10 | 1 | 10 |
| 2 | 8 | 2 | 20 | 4 | 40 |
| 3 | 9 | 3 | 30 | 4 | 40 |
| 4 | 10 | 4 | 40 | 5 | 50 |
| 5 | 10 | 4 | 40 | 5 | 50 |
| 6 | 12 | 6 | 60 | 5 | 50 |
| 7 | 13 | 7 | 70 | 6 | 60 |
| 8 | 19 | 8 | 80 | 9 | 90 |
| 9 | 20 | 9 | 90 | 9 | 90 |
| 10 | 21 | 10 | 100 | 10 | 100 |

# Appendix 9 Feature selection using LASSO and EN

Parametric feature selection methods were also used to select predictors and compared with the REF feature selection methods. LASSO and EN regression are regularisation technics that eliminate insignificant features, preserve correlated features, and are well suited to the high-dimensional feature vectors in our dataset [16]. Compared to LASSO, which tends to remove correlated features by assigning a non-zero coefficient to one feature while driving the coefficients of other correlated features to zero, EN tends to keep groups of correlated features by assigning small, non-zero coefficients to them [17]. For both methods, we used cross-validation to select appropriate values for the tuning parameter (lambda) and selected the values that minimised the mean cross-validated errors [18].

# Appendix 10 Example of syntax for REWIRED framework

The input data frame called ‘data23’ having 519 rows and 199 columns. Each row contained the observations of one of the 519 small geographic areas (sa2). For each sa2, there were 199 variables, including a sa2 code called ‘sa2’; a continuous outcome variable called ‘sir’ representing the lung cancer smoothed standardised incidence ratio for that sa2; and this continuous variable was categorised into a three-level categorical variable called ‘sirgrp’; and 196 area-level socio-environmental variables for that sa2, which were named as ‘se1’, ‘se2’, … ’se196’.

This example syntax just provides key functions that were used in key steps. More detailed syntax can be found in Github (https://github.com/KouKouAlison/REWIRED-Framework/blob/main/REWIRED%20Framework%20for%20lung%20cancer%20incidence%20in%20Queensland.R).

#############################

# Step 1 - Missing data imputation #

#############################

# library(caret)

# This step imputes the missing data for all the predictors

# Impute the missing values using bagging

set.seed(7777)

bagMissing <- preProcess(data23, method = "bagImpute")

data23_imputed <- predict(bagMissing, newdata = data23)

########################

# Step 2 - Variable selection #

########################

# This step selected 13 variables using the RFE method. Selecting variables using LASSO and EN methods in the comparative analysis were not included in this example.

# Force the number of features less than 15 #

subsets <- seq(2, 15, by=1)

set.seed(7777)

#Controlling the Feature Selection Algorithms

rfeCtrl <- rfeControl(functions = rfFuncs,

method = "boot",

rerank = TRUE,

verbose = FALSE)

rfProfile <- rfe(x=data23[,4:199],

y=data23[,1], #first column is the categorical outcome ‘sirgrp’

sizes = subsets,

rfeControl = rfeCtrl,

metric="Kappa")

features_rfe <- rfProfile$optVariables # model with 13 features has the highest Kappa

data23_ref <- data23 [, c("category", features_rfe)]

#####################################

# Step 3 - Split into training and testing sets #

#####################################

# This step split dataset into training and testing sets

set.seed(6789)

trainIndex <- createDataPartition(data23$sirgrp, p=0.8, list = FALSE)

trainingSet_rfe <- data23_rfe[trainIndex,]

testSet <- data23[-trainIndex,]

#####################################

# Step 4 - Training the random forest model #

#####################################

# This step trained the random forest model using k-fold cross validation sampling or bagging sampling method.

# Option 1 - Define the train control for cross-validation

ctrl_cv <- trainControl(

method = "cv", # k-fold cross-validation

number = 10, # 10-fold CV

savePredictions = "final",

classProbs = TRUE,

sampling = "up", # remove this if you have used SMOTE to oversample the minority groups

summaryFunction = multiClassSummary # multi-class summary function for comprehensive evaluation

)

# Option 2 - Define the training control options for bagging (bootstrap aggregating)

ctrl_boot <- trainControl(

method = "boot",

number = 100,

savePredictions = "final",

classProbs = T,

sampling = "up" #remove this if have used smote to oversampled the minority groups

)

# Define the parameter grid, including nodesize and mtry

tuneGrid <- expand.grid(mtry = seq(2, 13, by=1), nodesize = c(1, 5, 10, 15, 20))

# Train the model using k-fold CV sampling

set.seed(7777)

rf_cv_rfe <- train(sirgrp ~ .,

data = trainingSet_rfe,

method = customRF,

trControl = ctrl_cv,

tuneGrid = tuneGrid,

metric="Kappa")

# Train the random forest model using bagging sampling

set.seed(8888)

rf_boot_rfe <- train(category ~ .,

data = trainingSet_rfe,

method = "rf",

trControl = ctrl_boot,

tuneGrid = tuneGrid,

metric="Kappa")

##########################################

# Step 5 - Check model performance using test set #

##########################################

# This step generates the confusion matrix (only for model 2 in this example) using the test set. Final model was selected based on the confusion matrix results.

#predict for test set using model 2

fitted_boot_rfe_select <- predict(rf_boot_rfe_select, testSet)

#generate confusion matrix for prediction

confusionMatrix(reference=testSet$ sirgrp, data=fitted_boot_rfe_select, mode="everything", positive = "Exceed")

###########################################################################

#Step 6 - Calculate the gap adjusted percentile rank for all the 8 predictors for the 519 sa2 #

###########################################################################

# This step generates the gap-adjusted percentile rank of each sa2 for each of the predictors. Predictors those are negatively associated with lung cancer incidence were reversely ranked.

#write a loop for the 8 predictors in model 2

for (v in featureorder) {

fmax=max(data_impfeature[[v]])

fmin=min(data_impfeature[[v]])

mm <- fmax-fmin

n <- 519

gap <- mm/(n-1)

for (i in 1:519) {

m = floor((data_impfeature[[v]][i]-fmin)/gap)+1

rank[[v]][i] = m/n*100

}

}

##########################################

# Step 7 - Generate the cancer vulnerability index #

##########################################

# This step generates the cancer vulnerability index for each sa2 by summing up the rank*weight for each predictor

for (i in 1:519) {

for (v in featureorder) {

feature.score=weight$impscore[weight$features == v]

feature.rank=rank[i,v]

index_gapadj[i,v] <- feature.score*feature.rank

}

}

index_gapadj$index <- rowSums(index_gapadj[, 4:11])

# Appendix 11 Lists of predictors included in model 1-4.

Model 1&3 – RFE predictors:

- Diabetes mellitus (ASR)
- Adequate fruit intake (ASR)
- High-income taxpayers with private health insurance (%)
- Bachelor’s degree (%)
- Unemployed (%)
- Median annual income-Superannuation and annuity ($)
- Highest year of school completed - Year 11 or equivalent (%)
- Marital status-Separated (%)
- Prevalence of smoking (ASR)
- Prevalence of asthma (ASR)
- Percentage of males aged 10-14 years old (%)
- Percentage of employed persons in financial and insurance services (%)
- Median annual own unincorporated business income ($)

Model 2&4 – RFE_selected predictors:

- Diabetes mellitus (ASR2)
- Adequate fruit intake (ASR)
- High-income taxpayers with private health insurance (%)
- Bachelor’s degree (%)
- Unemployed (%)
- Median annual income-Superannuation and annuity ($1000)
- Highest year of school completed-Year 11 or equivalent (%)
- Marital status-Separated (%)

# Appendix 12 Confusion matrix based on test set predicted by Model 2

| **Predicted** | **Actual** | | |
| --- | --- | --- | --- |
|  | Above | Average | Below |
| Above | 13 | 8 | 1 |
| Average | 11 | 29 | 7 |
| Below | 0 | 11 | 23 |

# References

1. Cramb SM, Mengersen KL, and Baade PD, *Atlas of Cancer in Queensland: geographical variation in incidence and survival, 1998 to 2007*. 2011, Viertel Centre for Research in Cancer Control, Cancer Council Queensland.

2. Cramb, S.M., K.L. Mengersen, and P.D. Baade, *Developing the atlas of cancer in Queensland: methodological issues.* International Journal of Health Geographics, 2011. **10**: p. 1-11.

3. Cramb, S.M., *Spatio-temporal modelling of cancer data in Queensland using Bayesian methods*. 2015, Queensland University of Technology.

4. Kang, S., et al., *Making the most of spatial information in health: a tutorial in Bayesian disease mapping for areal data.* Geospatial health, 2016. **11**(2): p. Article number: 428 190-198.

5. Earnest, A., S. Cramb, and N. White, *Disease mapping using Bayesian hierarchical models*. 2013, Wiley Online Library. p. 221-239.

6. Riebler, A., et al., *An intuitive Bayesian spatial model for disease mapping that accounts for scaling.* Statistical methods in medical research, 2016. **25**(4): p. 1145-1165.

7. Duncan, E.W., et al., *Development of the Australian Cancer Atlas: spatial modelling, visualisation, and reporting of estimates.* International journal of health geographics, 2019. **18**: p. 1-12.

8. Lawson, A.B., W.J. Browne, and C.L.V. Rodeiro, *Disease mapping with WinBUGS and MLwiN*. Vol. 11. 2003: John Wiley & Sons.

9. Lee, D., *CARBayes: an R package for Bayesian spatial modeling with conditional autoregressive priors.* Journal of Statistical Software, 2013. **55**(13): p. 1-24.

10. *Australian Cancer Atlas 2.0 (*[*https://atlas.cancer.org.au*](https://atlas.cancer.org.au)*). Cancer Council Queensland and Queensland University of Technology. Version 05-2024. Accessed [give date]*.

11. BreastScreen Queensland. *Find a location*. 2024 [cited 2024 July]; Available from: <https://www.breastscreen.qld.gov.au/find-a-location>.

12. Stehman, S.V., *Selecting and interpreting measures of thematic classification accuracy.* Remote sensing of Environment, 1997. **62**(1): p. 77-89.

13. Agresti, A. and B.A. Coull, *Approximate is better than “exact” for interval estimation of binomial proportions.* The American Statistician, 1998. **52**(2): p. 119-126.

14. Viera, A.J. and J.M. Garrett, *Understanding interobserver agreement: the kappa statistic.* Fam med, 2005. **37**(5): p. 360-363.

15. Saah, A.J. and D.R. Hoover, *“Sensitivity” and “specificity” reconsidered: the meaning of these terms in analytical and diagnostic settings*. 1997, American College of Physicians.

16. Tibshirani, R., *Regression shrinkage and selection via the lasso.* Journal of the Royal Statistical Society Series B: Statistical Methodology, 1996. **58**(1): p. 267-288.

17. Zou, H. and T. Hastie, *Regularization and variable selection via the elastic net.* Journal of the Royal Statistical Society Series B: Statistical Methodology, 2005. **67**(2): p. 301-320.

18. Maharana, A. and E.O. Nsoesie, *Use of deep learning to examine the association of the built environment with prevalence of neighborhood adult obesity.* JAMA network open, 2018. **1**(4): p. e181535-e181535.
