# Supplementary material for: Generation of machine-learning derived cancer vulnerability indicator to determine the spatial burden of cancer outcomes
Source: PLoS One. 2026 Feb 20;21(2):e0319539. doi: 10.1371/journal.pone.0319539 (PMC12923003; doi:10.1371/journal.pone.0319539)
Supplement: S2 File — (DOCX) [file pone.0319539.s002.docx]

**Supporting Information S2. list of area-level datasets and direct URLs.**

1. ABS sourced - Population & People (SA2) 2011-2019. <https://data.aurin.org.au/dataset/au-govt-abs-abs-data-by-region-pop-and-people-asgs-sa2-2011-2019-sa2-2016>.

2. ABS sourced - Income (Including Government Allowances) (SA2) 2011-2019. <https://data.aurin.org.au/dataset/au-govt-abs-abs-data-by-region-income-asgs-sa2-2011-2019-sa2-2016>.

3. ABS sourced - Health & Disability (SA2) 2011-2018. <https://data.aurin.org.au/dataset/au-govt-abs-abs-data-by-region-health-and-disability-asgs-sa2-2011-2018-sa2-2016>.

4. ABS sourced - Family & Community (SA2) 2011-2018. <https://data.aurin.org.au/dataset/au-govt-abs-abs-data-by-region-family-and-community-asgs-sa2-2011-2018-sa2-2016>.

5. ABS sourced - Education & Employment (SA2) 2011-2019. <https://data.aurin.org.au/dataset/au-govt-abs-abs-data-by-region-education-and-employment-asgs-sa2-2011-2019-sa2-2016>.

6. PHIDU sourced - Prevalence of Selected Health Risk Factors - Adults (PHA) 2017-2018. <https://data.aurin.org.au/dataset/tua-phidu-phidu-estimates-risk-factors-adults-pha-2017-18-pha2016>.

7. PHIDU sourced - Prevalence of Chronic Diseases (PHA) 2017-2018. <https://data.aurin.org.au/dataset/tua-phidu-phidu-estimates-chronic-disease-pha-2017-18-pha2016>.

8. NHSD sourced – Location of health facilities. https://data.aurin.org.au/dataset/healthdirect_nhsd_services_directory_2023.
